# Stability, Content of Bioactive Compounds and Antioxidant Activity of Emulsions with Propolis Extracts during Simulated In Vitro Digestion

**DOI:** 10.3390/foods13050779

**Published:** 2024-03-02

**Authors:** Lucio González Montiel, Arely León-López, Adelfo García-Ceja, Melitón Jesús Franco-Fernández, Elizabeth Pérez-Soto, Antonio de Jesús Cenobio-Galindo, Rafael G. Campos-Montiel, Gabriel Aguirre-Álvarez

**Affiliations:** 1 Instituto de Tecnología de los Alimentos, Universidad de la Cañada, Teotitlán de Flores Magón 68540, Oaxaca, Mexico; luciogonzalez@unca.edu.mx; 2 TecNM Campus Venustiano Carranza, Av. Tecnológico S/N, Col. el Huasteco, Ciudad Lázaro Cárdenas, Puebla 73049, Mexico; arely.leon@vcarranza.tecnm.mx (A.L.-L.);; 3 Instituto de Ciencias Agropecuarias, Universidad Autónoma del Estado de Hidalgo, Av. Universidad Km 1 Rancho Universitario, Tulancingo 43600, Hidalgo, Mexico; mfranco@uaeh.edu.mx (M.J.F.-F.); epsoto@uaeh.edu.mx (E.P.-S.); antonio_cenobio@uaeh.edu.mx (A.d.J.C.-G.); rcampos@uaeh.edu.mx (R.G.C.-M.)

**Keywords:** total phenols, total flavonoids, antioxidant activity, ABTS, DPPH, propolis

## Abstract

The objective in this work was the evaluation of the stability and content of bioactive compounds (total phenols and total flavonoids) and antioxidant activity of emulsions of ethanolic extracts of propolis obtained by ultrasound, during simulated in vitro digestion. The emulsions prepared with propolis extracts were evaluated on certain properties: their emulsion efficiency, stability (zeta potential, particle size, electrical conductivity), content of bioactive compound (total phenolics and total flavonoids), antioxidant activity and their behavior during simulated in vitro digestion. Based on the total phenol content, an emulsification efficiency of 87.8 ± 1.9% to 97.8 ± 3.8% was obtained. The particle size of the emulsions was 322.5 ± 15.33 nm to 463.9 ± 33.65 nm, with a zeta potential of −31.5 ± 0.66 mV to −28.2 ± 1.0 mV and electrical conductivity of 22.7 ± 1.96 µS/cm to 30.6 ± 0.91 µS/cm. These results indicate good emulsion stability. During simulated in vitro digestion, the content of bioactive compounds (total phenolics, total flavonoids) and antioxidant activity were affected during 77 days of storage at 4 °C. It was concluded that the emulsion process fulfills the function of protecting the bioactive compounds and therefore their biological activity.

## 1. Introduction

During the last decades, interest in the study, characterization, use and application of natural products has increased because of the benefits they provide to health. Propolis is considered a natural, resinous substance that bees (*Apis mellifera*) process from secretions of buds, stems and bark of a great diversity of plants, shrubs and trees. These resinous secretions are mixed by bees with wax, pollen and enzymes, obtaining a material with a sticky, rubbery and balsamic appearance, of different shades of color, ranging from yellow, green, red, brown to black, with a strong, aromatic, bitter and spicy flavor [1,2,3]. The physicochemical composition of propolis depends on factors like vegetation, type of flowering, species of bees, time of year, geographical area, as well as climatic–environmental conditions [4,5]. Propolis is composed of 50% resins, 30% wax, 10% essential oils, 5% pollen and 5% of bioactive compounds.

Approximately 300 bioactive compounds have been identified in propolis, including phenols and flavonoids, aromatic and aliphatic acids and their esters, terpenes, tannins, lignans, carbohydrates, vitamins, enzymes and minerals [6,7,8]. Propolis has been used for treatments of different health problems [9] due to its beneficial health properties, such as; antioxidant, antibacterial, antifungal, anthelmintic, antiviral, anti-inflammatory, immunomodulatory, hepatoprotective, anti-allergic, healing and antitumor properties, among others [3,4,9,10]. 

Related to these bioactive properties of propolis, it is necessary to carry out more studies to boost the production of propolis and investigate some aspects such as the chemical composition, content of bioactive compounds and biological activity of propolis from various regions of Mexico. There are several protocols and methods for in vitro digestion. Approximately 90% of papers related to in vitro systems use static models [11]. These models use a series of discontinuous recipients with unique and constant biochemical conditions such as pH, temperature, enzyme content and concentration, electrolytes and biliary salts [12]. It is important to note that no special technology is required to carry out the experimental work in labs, as only shakers, water baths, incubators with orbital agitation and magnetic stirrers are needed [13].

Saliba et al. [14] and Alencar et al. [15] emphasized that many of the bioactive compounds present in propolis extracts could be affected during simulated in vitro digestion mainly by gastrointestinal conditions, causing isomerization and hydrolysis of phenolic compounds, decreasing their biological properties. According to Alegría et al. [16], the static models are commonly utilized during gastric and intestinal steps due to their limited digestion. These models are used for analysis of simple foods as well as purified food components. In this method, there is a good control of parameters (pH, digestion time, type of enzyme) to obtain reliable results. Due to the aforementioned, several methodologies have been developed for the protection of bioactive compounds in propolis extracts ranging from encapsulation, microencapsulation, emulsions, microemulsions and nanoemulsions, making use of different protection agents, among which stand out: dextrins, maltodextrins, gums, starches, modified starches, proteins, alginates, oils, carbohydrates and polymers, among others [14,15,17,18,19,20]. The emulsion process protects the bioactive compounds of propolis from gastrointestinal conditions. Also, it could avoid the interaction of environmental factors such as light, temperature, humidity, radiation and oxygen reactions. Protection also includes the reduction of evaporation, increments of solubility, functionality and bioavailability. Among the most important advantages of the emulsions prepared in this research is their application within the food industry due to their capacity to solubilize in water, as well as the capacity to mask the unpleasant taste of propolis and the smell of native propolis [21,22,23,24]. The importance of the incorporation of encapsulated propolis in several formulations to be applied to medicines, cosmetics and food as a functional additive is well documented [4,25,26,27]. 

The relationship between diet and human health could be analyzed through the bioaccessibility in vitro evaluation [28,29]. Bioaccessibility can be defined as a component of bioavailability, in other words, the proportion of food compounds that reach the systemic circulation after ingestion. It is an important concept in human health and nutrition, related to the compounds released from the food matrix in the gastrointestinal tract for their absorption in the human body [30,31]. During gastrointestinal processes, the gastric juices break down the food and the bioactive compounds in the stomach and move across the intestinal lining and into the bloodstream as bioavailable molecules with positive effects in the body [32,33]. However, the non-bioaccessible fractions of phenolic compounds are transported to the colon, where they can be absorbed by the epithelium. These phenolic compounds are metabolized by colon bacteria that transform diet polyphenols into simple phenols with higher biological activity [34]. The pH and biliary salt parameters of the intestinal environment can modify the chemical structures of bioactive compounds, resulting in new molecules decreasing bioavailability and biological activity [35,36]. The objective of this investigation was to study the stability and content of bioactive compounds and antioxidant activity of emulsions of propolis extracted with ethanol and ultrasound, during simulated in vitro digestion.

## 2. Materials and Methods

### 2.1. Propolis Sources

Propolis was collected from three different sites: Teotitlán de Flores Magón (18°08′00″ N 97°05′00″ W), San Pedro Ocopetatillo (18°12′00″ N 96°55′00″ W) and San Jerónimo Tecóatl (18°10′00″ N 96°55′00″ W), belonging to the Mazatec area of Oaxaca state, Mexico. The samples were collected at apiaries with the support of two beekeepers’ organizations: “Miel Néctar Mazateco” and “Sociedad de Apicultores de Eloxochitlán”. The samples were transported to the University of the Cañada in hermetically closed containers, protected from light, and stored at −30 °C until use.

### 2.2. Extraction Method and Preparation of Extracts

The preparation of extracts was carried out according to the methodology of Osés et al. [37], with some modifications. The frozen propolis (−30 °C) was pulverized in a mill (Jia-wanshun, Model HC-2000Y, Shenzhen, China), and extraction was carried out from the powdered propolis. Ultrasound was applied to samples in an ultrasonic bath (Branson 3510, Marshall Scientific, Hampton, NH, USA) for 30 min at a frequency of 42 kHz. Ethanol (85%) was used in a proportion of 1:30 *w*/*v*, at 20 °C. Subsequently, centrifugation was performed at 18,510 × *g* for 15 min at 4 °C to eliminate only non-extractable solids. The extraction solvent was concentrated in a rotary evaporator at 40 °C (Büchi R-215, Marshall Scientific). A vacuum oven at 40 °C was used to completely remove the solvent. The extracts were labeled as follows: Teotitlán (T), San Pedro (SP) and San Jerónimo (SJ). The samples were stored in amber glass containers at 4 °C until use. The content of total phenols, total flavonoids and antioxidant activity were determined by 2,2′-azino-bis (3-ethylbenzothiazoline-6-sulfonic acid) (ABTS+) and 1,1-diphenyl-2-picrylhydrazyl radical (DPPH+).

### 2.3. Preparation, Characterization and Stability of Emulsions

The emulsions were prepared according to Espino-Manzano et al. [38] and Seibert et al. [23] with some modifications. The composition of the emulsions was: 3% sorbitan monooleate (Span 80, Sigma-Aldrich, St. Louis, MO, USA), 5% pure propolis extract, 5% orange essential oil (W282510, Sigma-Aldrich, St. Louis, MO, USA), 7% polysorbate 80 (Tween 80, Sigma-Aldrich, St. Louis, MO, USA) and 80% ultrapure water (Milli-Q, Millipore, Sigma-Aldrich, St. Louis, MO, USA). The propolis extract, orange essential oil, Span 80 and Tween 80 were mixed with an ultrasonic processor (Ultrasonic Vibra-Cell VCX 130 Sonics, Newton, CT, USA) at a frequency of 20 kHz, at 80% amplitude. We applied 36 intervals of 50 s with 10 s of rest with a 6 mm diameter probe. Ultrapure water was added and 36 intervals of 50 s with 10 s of rest were applied. The mixture remained in an ice bath throughout the process. The emulsions were labeled (ET, ESP, and ESJ) and placed in amber glass bottles. Samples were refrigerated (4 °C) until analysis. To determine the particle size (nm), zeta potential (mV) and electrical conductivity (µs/cm) of the emulsions, a Zetasizer device (Nano-ZS2000 Model, Malvern Instruments Ltd., Malvern, Worcestershire, UK) was used, with 20 µL of the emulsion and 1980 µL of ultrapure water. The analysis was carried out after 0, 7, 14, 49, 56, 63, 70 and 77 days of storage. All measurements were carried out in triplicate. It is important to note that a control (without propolis) was also analyzed, in order to remove the possible antioxidant activity of all ingredients that composed the emulsion except propolis.

### 2.4. Encapsulation Efficiency

The emulsification efficiency was calculated according to Saliba et al. [14] with some modifications. The following equation was applied:

(1) $$Emulsion\;efficiency=\left(CTP\;of\;PE-CEP\right)/\left(CTP\;of\;PE\right)$$

where CTF of PE is the content of total phenols for pure extract and CEP is the content of total phenols in the emulsion. To break the emulsion, an ethanol–methanol solution (1:1 *v*/*v*) was added in a 1:1.2 *v*/*v* ratio (for each 1 mL of emulsion, 1.2 mL of the ethanol–methanol solution was added) and stirred vigorously for 30 min in the absence of light. Samples were centrifuged at 18,510 × *g* for 30 min at 4 °C, and the supernatant was used for analysis.

### 2.5. In Vitro Digestion of Emulsions with Propolis Extracts

Simulation of gastrointestinal digestion of emulsions with propolis extracts was carried out in vitro according to Saliba et al. [14] and Minekus et al. [39] with some modifications. Digestion was executed in two phases: (a) Gastric phase: 10 mL of each of the emulsions (ET, ESP and ESJ) and the control (distilled water) was used. A volume of 15 mL of gastric fluid (2000 U/mL of porcine pepsin in 0.3 M CaCl_2_ in 0.1 M HCl) was added, followed by the adjustment of the pH value with 6 M HCl up to 2. Samples were incubated at 37 °C in a shaking water bath for 120 min. (b) Intestinal phase: this phase began with the adjustment of the pH value up to 7 with 0.5 M sodium bicarbonate. Subsequently,10% pancreatic fluid (0.4 g of pancreatin and 2.5 g of bile salts in 100 mL of 0.1 M NaHCO_3_) was added. The mixture was incubated in a shaking water bath at 37 °C for 120 min followed by boiling for 4 min in a boiling water bath to inactivate enzymes. Samples were taken at the end of each of the phases (Gastric and Intestinal) and centrifuged (Hermle, Z 36 HK, Gosheim, Germany) at 18,510 × *g* for 10 min at 4 °C. Samples were subsequently stored in a refrigerator until analysis of total phenolics, flavonoids and antioxidant activity (ABTS+ and DPPH+).

### 2.6. Determination of Bioactive Compounds

#### 2.6.1. Quantification of Total Phenols

The quantification of phenols followed the Folin–Ciocalteu method described by Rababah et al. [40], with some modifications. For T, SP and SJ extracts, a dilution of propolis extract (1:1000 *w*/*v*) was performed using 85% ethanol. For ET, ESP and ESJ emulsions, 1.2 mL of ethanol–methanol was added per mL of emulsion. Samples were centrifuged at 18,510 × *g* for 15 min at 4 °C (Hermle, Z 36 HK, Gosheim, Germany). Aliquots (0.3 mL) of the supernatant were mixed with 1.5 mL of the 0.2 N Folin–Ciocalteu reagent in a test tube followed by incubation in the darkness at room temperature for 8 min. Then, 1.2 mL of 0.7 M Na_2_CO_3_ was added, homogenized and incubated at room temperature for 120 min in the darkness. The measurements of absorbance were carried out in a spectrophotometer (Jeanway 6715, Cole-Parmer, Vernon Hills, IL, USA) at 765 nm. Water blank was used as a control. The total phenolic content was determined from a gallic acid standard curve (0 to 100 mg/L). All samples were analyzed in triplicate, and the results are expressed as mg equivalents of gallic acid (mg GAE/100 g propolis extract and emulsion).

#### 2.6.2. Quantification of Total Flavonoids

The total flavonoid content followed the methodology described by Arvouet-Grand et al. [41]. Samples of propolis extract identified as T, SP and SJ were diluted (1:1000 *w*/*v*) with methanol. For the ET, ESP and ESJ emulsions, 1.2 mL of ethanol–methanol was added per mL of the emulsion. The samples were homogenized for solubilization with a Vortex. Then, samples were centrifuged at 18,510 × *g* for 15 min at 4 °C (Hermle, Z 36 HK, Gosheim, Germany). A 2% solution of aluminum chloride (AlCl_3_) in methanol was prepared. A mixture of 2 mL of the supernatant with 2 mL of AlCl_3_ was homogenized in a test tube and left to rest at room temperature for 20 min in the darkness. The absorbance of samples was measured in a spectrophotometer at 415 nm (Jenway 6715) using methanol as a blank. Quercetin was used to prepare the standard curve (0 to 100 mg/L). All samples were analyzed in triplicate, and the results are expressed in equivalent mg of quercetin/100 g of propolis extract and emulsion (mg QE/100 g of propolis extract and emulsion).

### 2.7. Antioxidant Activity

#### 2.7.1. ABTS Free Radical Inhibition Activity

ABTS inhibition was evaluated by using 2,2′-azino-bis (3-ethylbenzothiazoline-6-sulfonic acid) (ABTS) as reported by Pimentel-González et al. [42]. Samples T, SP and SJ extracts were diluted (1:1000 *w*/*v*) in 85% ethanol. For ET, ESP and ESJ emulsions, 1.2 mL of ethanol–methanol was added per mL of the emulsion. The extracts were centrifuged at 18,510× *g* for 15 min at 4 °C (Hermle, Z 36 HK, Gosheim, Germany). We prepared 20 mL of 7 mM ABTS+ stock solution (36 mg of ABTS reagent) with distilled water, and 10 mL of 2.45 mM potassium persulfate (5.83 mg of potassium persulfate in 10 mL of distilled water) were added. To generate ABTS+ free radicals, the mixture was kept under magnetic stirring for 24 h in absence of light. Prior to reading the samples, the ABTS+ reagent was standardized to an absorbance of 0.7 + 0.01 using 20% ethanol. An aliquot of 0.2 mL of the supernatant was placed in a test tube with 2 mL of standardized ABTS+. After homogenization, samples were stored at room temperature for 6 min in the darkness. The readings were taken at 734 nm in a spectrophotometer (Jenway 6715). The standard curve (0 to 100 mg/L) was prepared with gallic acid, and 20% ethanol was used as a blank. All samples were analyzed in triplicate, and the antioxidant activity is expressed as mg equivalents of gallic acid in 100 g of extract and emulsion.

#### 2.7.2. DPPH Free Radical Inhibition Activity

The determination of the antioxidant activity of DPPH (1,1-diphenyl-2-picrylhydrazyl radical) was carried out according to the methodology of Turkmen et al. [43]. Samples T, SP and SJ were diluted (1:1000 *w*/*v*) in methanol. For emulsions (ET, ESP and ESJ), 1.2 mL of ethanol–methanol was added per mL of the emulsion. The extracts were centrifuged at 18,510 × *g* for 15 min at 4 °C (Hermle, Z 36 HK, Gosheim, Germany), and 100 mL were prepared of a 0.2 mM DPPH stock solution (7.9 mg of DPPH making up to 100 mL with 80% methanol). To generate DPPH+ free radicals, the mixture was kept under magnetic stirring for 2 h in total darkness. Prior to readings, the DPPH+ reagent was standardized to an absorbance of 0.7 + 0.01 using 80% methanol. An aliquot of 2.5 mL of the standardized DPPH+ reagent was added to 0.5 mL of the supernatant. The samples were incubated in darkness for 1 h. Readings were taken at 517 nm in a spectrophotometer (Jenway 6715), and methanol was used as a control. All samples were analyzed in triplicate, and the antioxidant activity was expressed as mg equivalents of gallic acid in 100 g of extract and emulsion.

### 2.8. Statistical Analysis

The results are expressed as mean ± standard deviation and were statistically analyzed using SAS software version 9.22 for Windows (9.22, Institute Inc., Cary, NC, USA). A one-way analysis of variance and comparison of means by the Tukey test was applied with a level of significance of *p* < 0.05.

## 3. Results and Discussion

### 3.1. Bioactive Compounds and Antioxidant Activity of Propolis Extracts

#### 3.1.1. Total Phenols

Table 1 shows the results of the content of bioactive compounds and antioxidant activity of the propolis extracts obtained by ultrasound. The results from TPC, flavonoids and antioxidant activity were obtained subtracting the control results.

#### 3.1.2. Total Flavonoids

The total phenolic content was 33.32 g GAE/100 g for the T propolis extract, 32.83 g GAE/100 g for SP and 32.58 g GAE/100 g for SJ. There was a significant difference (*p* < 0.05) between the propolis extracts of T and SJ, but no significant difference (*p* > 0.05) was found between the SP and SJ treatments. Shakoury et al. [20] reported values greater than 40.20 g GAE/100 g of propolis extract from Iran. The authors stated that the particle size of the propolis influences the extraction process: the smaller the particle size, the better the interaction with the solvent and therefore the higher the yield. Green propolis from Brazil had high content of phenolic compounds (31.64 to 53.39 g GAE/100 g) improving the yield under ultrasound-assisted conditions [44]. On the other hand, Peixoto et al. [45] obtained a range of 17.36 to 26.22 g GAE/100 g in propolis extracts from two regions of Portugal. Also, Ozdal et al. [46] found maximum values of 19.96 g GAE/100 g in propolis samples from different regions of Turkey. These results are lower than those reported in the present investigation. Rivero-Cruz et al. [47] determined the content of total phenolics in ethanol extract of propolis from the state of Guanajuato, Mexico, and reporting a lower range from 16.76 to 24.63 g GAE/100 g. This result could be due to the type of vegetation as well as the geographical location of the municipalities.

As illustrated in Table 1, the flavonoid content ranged from 3.67 to 20.59 g QE/100 g. All extracts showed a significant difference (*p* < 0.05). The highest content was presented by the T extract and the lowest content was presented by the SJ extract. Recently, around 140 flavonoids have been reported as flavones, flavonols, flavanones, flavanonols, isoflavones, dihydroflavonols, isodihydroflavones, etc.; these represent about 50% of the weight of propolis and are directly related to the antimicrobial activity [6,7,9]. The total flavonoid content in propolis from Portugal was 4.39 to 6.79 g QE/100 g [45]. Ozdal et al. [46] reported values of 3.073 to 29.175 mg QE/100 g in propolis samples from different geographical regions of Turkey. It is important to emphasize that propolis is rich in bioactive compounds with polyphenols being the most abundant flavonoids. The content and composition of these components depend on various factors, such as vegetation, type of flowering, bee species, time of year, geographical area and climatic conditions [5,9].

#### 3.1.3. Antioxidant Activity

Table 1 shows that the antioxidant capacity (ABTS+) presented a significant difference (*p* < 0.05) in the three extracts, i.e., T, SP and SJ, with values of 8.69, 14.31 and 14.01 g GAE/100 g, respectively. DPPH+ showed results of 21.66, 19.83 and 21.63 g GAE/100 g for T, SP and SJ extracts, respectively. Rivero-Cruz et al. [47] reported that Mexico is a megadiverse country. It produces a wide variety of propolis; however, studies are needed to validate or corroborate its functional properties. Propolis from Mexico is an important source of bioactive compounds with excellent antioxidant activity [48]. Okińczyc et al. [49] reported a maximum antioxidant activity (DPPH+) of 6.44 g GAE/100 g in propolis from Eurasia. These authors emphasized that flavonoids are not directly related to this property; however, the results of this research could indicate the presence of phenolic compounds (total phenolics, flavonoids, among other non-flavonoid phenolics). The results for DPPH+ were higher than those of ABTS+, which could be mainly due to the phytochemical composition of propolis, the solubility of the chemical compounds and the polarity of the solvents used [7].

### 3.2. Stability of Emulsions

#### 3.2.1. Emulsion Efficiency

Based on the quantification of total phenols, the efficiency of the emulsions with 5% propolis extracts was 87.8% for the emulsion with ET propolis, 94.3% for the emulsion with ESP propolis and 97.8% for the emulsion with ESJ propolis. There is little or no scientific information related to the preparation of propolis emulsions similar to our formulations. However, there are some publications related to the encapsulation and nanoencapsulation of propolis, using different polymers. Shakoury et al. [20] reported an encapsulation efficiency of 64.3 to 84% in propolis nanoparticles with whey protein. During the encapsulation process of Brazilian red propolis, the drying temperature decreased the total phenol content by 37%. Therefore, a maximum encapsulation efficiency of 63% was obtained [14]. Ligarda-Samanez et al. [19] reported low encapsulation efficiency from 20.93 to 58.64% when maltodextrins, tara gum and modified native potato starch were used as wall material in spray drying encapsulation. Seibert et al. [23] reported nanoemulsions with 1% propolis extract and 5% corn oil. In this project, formulations of 5% propolis extract and 5% orange essential oil were applied. It is worth mentioning that these authors did not analyze the efficiency of emulsion. Double emulsions of propolis with ethylcellulose showed an emulsion efficiency of 80.3% [50]. The use of rice (90.2%) and pea protein (89.25%) presented excellent efficiency in the preparation of nanocapsules with propolis These results were very similar to those reported by Jansen-Alves et al. [51].

#### 3.2.2. Particle Size

**Figure 1:**
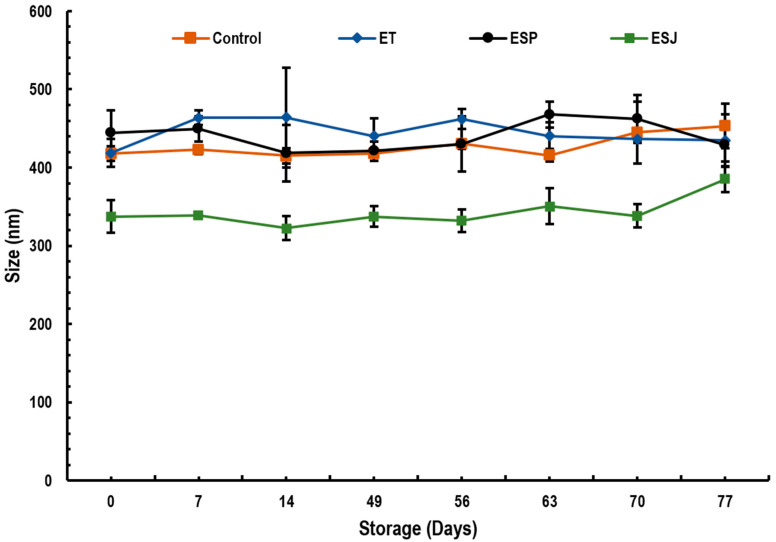
Droplet size stability (nm) of emulsions with propolis extract, during storage for 77 days at 4 °C. ET = emulsion with propolis extract from Teotitlán de Flores Magón, ESP = emulsion with propolis extract from San Pedro Ocopetatillo, ESJ = emulsion with propolis extract from San Jerónimo Tecoalt.

Figure 1 shows the results of the drop size of emulsions containing 5% propolis extract from three municipalities in the Oaxaqueña Cañada. The drop size ranged between 322.5 and 463.9 nm. For this reason, they cannot be defined as nanoemulsions since only monodisperse dispersions with a drop size between 20 and 200 nm in diameter are known as nanoemulsions. Seibert et al. [23] reported droplet sizes smaller than 65.2 nm in propolis emulsions using corn oil and 1% propolis extract. Ligarda-Samanez et al. [19] reported a maximum particle size of 430.8 nm in propolis microcapsules with a mixture of maltodextrins, tara gums and modified potato starch. The use of ovalbumin as a wall material generated microcapsules of 6.72 to 6.87 µm. These values were higher compared with those of this research [18]. Similar data were reported by Baysan et al. [17], with values from 1.42 to 52.75 µm using a mixture of carbohydrates and proteins. They explained that the wall material plays an important role in the particle size as well as the drying conditions of propolis microcapsules. Paulo et al. [50] mentioned that the amount of propolis extract is not related to the particle size; however, the conditions for preparing the emulsions, such as type of emulsifiers, emulsification time, emulsification speed, time and speed of solvent evaporation, type of solvent, etc., have a direct influence [52].

#### 3.2.3. Zeta Potential

**Figure 2:**
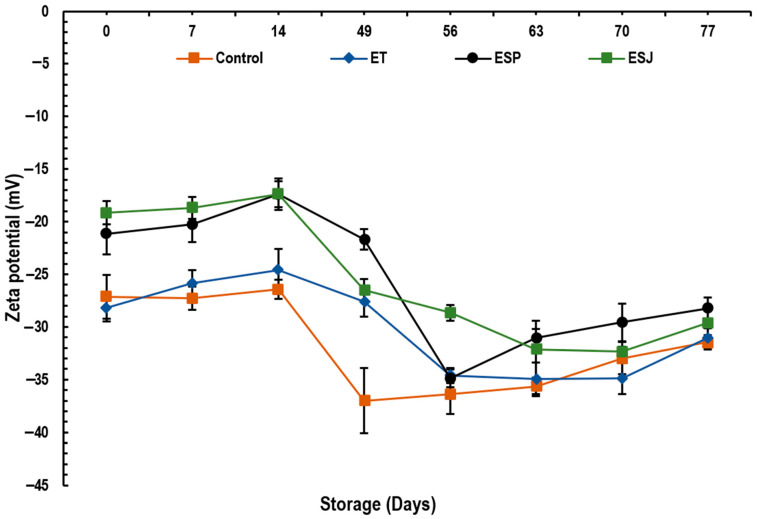
Zeta potential (mV) of emulsions with propolis extract, during storage for 77 days at 4 °C. ET = emulsion with propolis extract from Teotitlán de Flores Magón, ESP = emulsion with propolis extract from San Pedro Ocopetatillo, ESJ = emulsion with propolis extract from San Jerónimo Tecoalt.

In Figure 2, the results of the zeta potential emulsions containing 5% propolis extract from three municipalities in the Oaxaqueña Cañada are presented. No significant difference (*p* > 0.05) was observed between the samples at the end of storage. The values for Control, ET, ESP and ESJ were −31.5, −31.0, −28.2 and −29.6 mV, respectively. An emulsion is considered stable as long as its zeta potential is greater than +30 mV [23,52]. The authors reported values of −11.53 to −12.77 mV reflecting low stability of their emulsions. Based on the zeta potential (−27.2 to −38.7 mV), propolis microcapsules with maltodextrins, tara gum and modified starch are considered to have medium stability [19]. However, Shakoury et al. [20] considered that propolis nanocapsules with whey protein are very stable, presenting values of +27.5 to −24.61 mV. Low values of zeta potential (−6.16 to 0.42 mV) in propolis microcapsules using proteins as protective material can be attributed to the poor formation of cross-links due to the poor denaturation of proteins during the encapsulation process [17]. Therefore, it is confirmed that the emulsions of this research presented good stability during 77 days of storage at 4 °C.

#### 3.2.4. Electrical Conductivity

**Figure 3:**
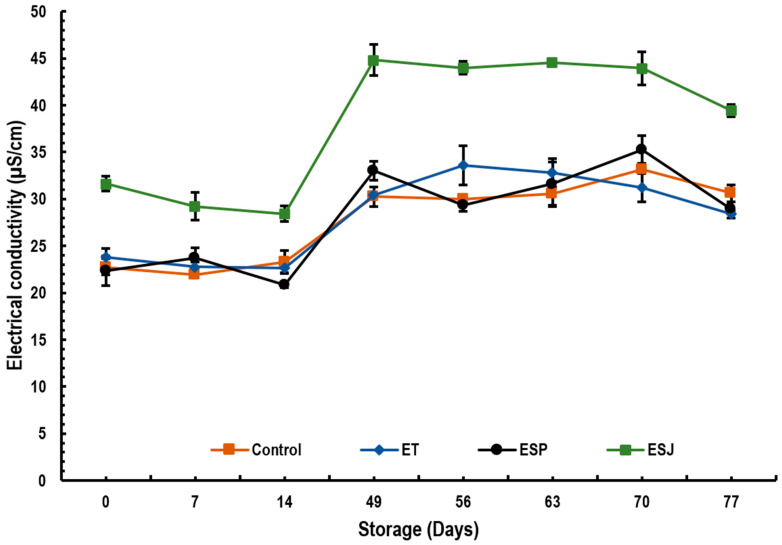
Electrical conductivity (µS/cm) of emulsions with propolis extract, during storage for 77 days at 4 °C. ET = emulsion with propolis extract from Teotitlán de Flores Magón, ESP = emulsion with propolis extract from San Pedro Ocopetatillo, ESJ = emulsion with propolis extract from San Jerónimo Tecoalt.

Figure 3 shows the results of the electrical conductivity of emulsions containing 5% propolis extract from three municipalities in the Oaxaqueña Cañada. The control on day 0 showed an electrical conductivity of 22.7, and at the end of storage it was 30.6 µS/cm. The ET emulsion went from 23.7 to 28.4 µS/cm, ESP from 22.4 to 28.9 µS/cm and ESJ from 31.63 to 39.4 µS/cm. Seibert et al. [23] reported electrical conductivity values of 36.47 µS/cm in 1% propolis nanoemulsions.

### 3.3. Behavior of Bioactive Compounds from Emulsions during Simulated In Vitro Digestion

#### 3.3.1. Total Phenols

Table 2 shows the results of the total phenolic content during the simulated in vitro digestion of the emulsions with 5% propolis extracts from 0 to 77 days of storage at 4 °C. During the gastric phase (GP), the content of total phenols on day 0 ranged from 1.48 to 1.53 g GAE/100 g. No significant difference was found (*p* > 0.05) between the emulsions with 5% propolis extract after 77 days of storage at 4 °C. The total phenol content ranged from 1.31 to 1.42 g GAE/100 g. These results could indicate that the total phenol content was not affected by the gastric conditions. During the intestinal phase (IP) on day 0, an increase in the total phenolic content was observed from 1.50 to 1.76 g GAE/100 g. A similar behavior was observed on day 77 of storage at 4 °C, where the total phenolic content ranged from 1.56 to 1.81 g GAE/100 g. Simulated in vitro digestion is considered a useful model test to understand the behavior during human in vitro digestion, although other factors may influence it [46]. The gastrointestinal conditions such as pH and enzyme content can react with phenolic compounds causing a decrease associated with the isomerization and hydrolysis of the compounds [14]. Oil–water emulsions used in the protection of fat-soluble extracts have better bioavailability due to the high solubility of these compounds [53]. Boyraci et al. [54] reported a decrease from 91 to 87% of phenolic compounds in propolis extracts during gastrointestinal digestion. Jansen-Alves et al. [18] reported a reduction in phenolic compounds during the gastric phase of microcapsules with propolis extracts, possibly due to the enzymatic attack suffered by the proteins used as wall material. The bioavailability of phenolic compounds in nanocapsules with Brazilian red propolis extract was affected by 45 to 80% during in vitro digestion [15]. Vlaicu et al. [28] mentioned that the bioaccessibility and bioavailability of bioactive compounds depend on several factors (food matrix, food ingredients and digestive process) to exert their functional and physiological effects. These results indicate that the emulsion helps protect the bioactive compounds and consequently enables better bioaccessibility of the compounds [36]. Therefore, the bioavailability of bioactive compounds can be increased, as can their beneficial effects on health.

#### 3.3.2. Total Flavonoids

The content of total flavonoids in the emulsions on day 0 after gastric digestion ranged from 2.15 to 1.19 g QE/100 g of emulsions with 5% propolis extract presenting a significant difference (*p* < 0.05) in the ET emulsions compared to ESP and ESJ treatments (see Table 2). These results are mainly due to the composition and place of origin of the propolis. Among treatments, there was no significant difference (*p* > 0.05) between day 0 and day 77 of storage at 4 °C in the content of total flavonoids during the gastric phase (GP). The flavonoids were not affected by gastric conditions or storage time. Wojtunik-Kulesza et al. [55] reported that during intestinal enzymatic action, there could be an increase in bioactive compounds. The ESJ emulsion presented the lowest content (0.19 g QE/100 g) and a significant difference (*p* < 0.05) from ET and ESP samples. At the end of storage (77 days at 4 °C) there was no significant difference (*p* > 0.05) in the values of the gastric phase. This could indicate that intestinal conditions do not affect the flavonoids of the emulsions containing 5% extract of propolis. At the end of gastrointestinal digestion, the bioaccessibility of flavonoids from nanoencapsulated Brazilian red propolis increased from 15 to 17% [15]. Ozdal et al. [46] reported an increase in total flavonoid content in propolis extracts at the end of intestinal digestion. Yesiltas et al. [56] found that after the gastrointestinal digestion of propolis extracts, the bioaccessibility of total flavonoids was reduced to 0.2%.

#### 3.3.3. Antioxidant Activity

Table 3 shows the results on the antioxidant activity (ABTS+ and DPPH+) during the in vitro digestion of emulsions with 5% propolis extract from three municipalities in the Oaxaqueña Cañada. During the gastric phase, a significant difference (*p* < 0.05) was observed between the emulsions, mainly associated to the place where the propolis was collected. The emulsion with the lowest antioxidant activity by the ABTS+ method was the GPET emulsion with 0.40 ± 0.06 g GAE/100 g. It was observed that at the end of storage (77 days at 4 °C) there was no significant difference (*p* > 0.05) for each of the emulsions. This behavior is similar to that of the total phenolic content (Table 2). The antioxidant activity by the DPPH+ method was greater than ABTS+, and no significant difference (*p* > 0.05) was found in any of the emulsions in the gastric and intestinal phases. This behavior occurred on day 0 up to 77 days of storage at 4 °C. Therefore, during simulated in vitro digestion, the compounds responsible for antioxidant activity were not affected by gastric and intestinal conditions, and the emulsions remained stable during this time. The antioxidant activity in microencapsulated Brazilian red propolis extracts decreased significantly during simulated gastrointestinal digestion [14]; these results are very similar to those reported by Alencar et al. [15] in encapsulations with Brazilian red propolis extract with different wall materials. Gomes Sá et al. [57] reported that microcapsules with Brazilian red propolis extract (different wall material and three encapsulation methods) had good bioavailability, and the antioxidant activity (ABTS+) was not affected after 108 days of storage. It is important to highlight that there are few investigations related to the stability (storage time) of emulsions with propolis extracts, as well as their evaluation of antioxidant activity during simulated in vitro digestion.

## 4. Conclusions

The propolis extracts obtained by ultrasound showed a high content of phenolic compounds (total phenols and total flavonoids) as well as excellent antioxidant activity. The emulsification process resulted in an excellent emulsification efficiency, and the emulsions presented very good stability at the end of storage time. During the simulated in vitro digestion, the emulsion process fulfilled the function of protecting the bioactive compounds and therefore their biological activity. We recommended evaluating the functionality and bioaccessibility of the investigated emulsions, including their incorporation in different formulations for their application in the food, cosmetics and drug industries, among others.

## Figures and Tables

**Table 1:** Content of bioactive compounds and antioxidant activity of propolis extracts from three municipalities of the Oaxaqueña Cañada.

Extract	Total Phenols g GAE/100 g	Total Flavonoids g QE/100 g	ABTS+ g GAE/100 g	DPPH+ g GAE/100 g
T	33.32 ± 0.13 ^a^	20.59 ± 0.276 ^a^	8.69 ± 0.87 ^c^	21.66 ± 0.78 ^a^
SP	32.83 ± 0.44 ^ab^	5.17 ± 0.99 ^b^	14.31 ± 0.94 ^a^	19.83 ± 0.51 ^b^
SJ	32.58 ± 0.30 ^b^	3.67 ± 0.17 ^c^	14.01 ± 0.86 ^b^	21.63 ± 0.51 ^a^

The results are expressed as mean ± standard deviation. Different lowercase letters in the columns represent significant differences (*p* < 0.05) based on a comparison of means by the Tukey test. T = propolis extract from Teotitlán de Flores Magón, SP = propolis extract from San Pedro Ocopetatillo, SJ = propolis extract from San Jerónimo Tecoalt.

**Table 2:** Content of total phenolics and total flavonoids during simulated in vitro digestion of emulsions with propolis extracts.

Phase	Total Phenols g GAE/100 g		Total Flavonoids g QE/100 g	
	Day 0	Day 77	Day 0	Day 77
GPET	1.48 ± 0.37 ^abA^	1.42 ± 0.17 ^abA^	1.19 ± 0.35 ^aB^	1.10 ± 0.32 ^aB^
GPESP	1.53 ± 0.18 ^abA^	1.40 ± 0.10 ^abA^	2.63 ± 0.03 ^bcdB^	0.243 ± 0.03 ^bcdB^
GPESJ	1.51 ± 0.17 ^abA^	1.31± 0.72 ^bA^	2.15 ± 0.03 ^cdB^	0.199 ± 0.02 ^dB^
IPET	1.65 ± 0.12 ^abA^	1.59 ± 0.25 ^abA^	0.92 ± 0.85 ^abcB^	0.97 ± 0.90 ^abB^
IPESP	1.76 ± 0.10 ^aA^	1.81 ± 0.11 ^aA^	0.25 ± 0.01 ^bcdB^	0.32 ± 0.02 ^bcdB^
IPESJ	1.50 ± 0.39 ^abA^	1.56 ± 0.54 ^abA^	0.16 ± 0.05 ^dB^	0.21 ± 0.07 ^cdB^

The results are expressed as mean ± standard deviation. Different lowercase letters in the columns represent a significant difference (*p* < 0.05) with a comparison of means by Tukey’s test. Different capital letters in the rows represent a significant difference (*p* < 0.05) with a comparison of means by Tukey’s test. GPET = gastric phase emulsion with propolis extract from Teotitlán de Flores Magón, GPESP = gastric phase emulsion with propolis extract from San Pedro Ocopetatillo, GPESJ = gastric phase emulsion with propolis extract from San Jerónimo Tecoalt, IPET = intestinal phase emulsion with extract of propolis from Teotitlán de Flores Magón, IPESP = intestinal phase emulsion with propolis extract from San Pedro Ocopetatillo, IPESJ = intestinal phase emulsion with propolis extract from San Jerónimo Tecoalt.

**Table 3:** Antioxidant activity during simulated in vitro digestion of emulsions with propolis extracts.

Treatment	ABTS+ g GAE/100 g		DPPH+ g GAE/100 g	
	Day 0	Day 77	Day 0	Day 77
GPET	0.40 ± 0.06 ^bcA^	0.36 ± 0.026 ^cA^	0.85 ± 0.046 ^aB^	0.85 ± 0.068 ^aB^
GPESP	0.61 ± 0.014 ^aA^	0.57 ± 0.019 ^abA^	0.70 ± 0.042 ^aB^	0.70 ± 0.064 ^aB^
GPESJ	0.70 ± 0.014 ^aA^	0.65 ± 0.003 ^aA^	0.75 ± 0.049 ^aB^	0.74 ± 0.068 ^aB^
IPET	0.38 ± 0.012 ^cA^	0.43 ± 0.003 ^bcA^	1.00 ± 0.045 ^aB^	0.873 ± 0.074 ^aB^
IPESP	0.64 ± 0.029 ^aA^	0.56 ± 0.025 ^abA^	0.68 ± 0.023 ^aB^	0.74 ± 0.048 ^aB^
IPESJ	0.66 ± 0.030 ^aA^	0.57 ± 0.015 ^abA^	0.83 ± 0.029 ^aB^	0.82 ± 0.049 ^aB^

The results are expressed as mean ± standard deviation. Different lowercase letters in the columns represent a significant difference (*p* < 0.05) with a comparison of means by Tukey’s test. Different capital letters in the rows represent a significant difference (*p* < 0.05) with a comparison of means by Tukey’s test. GPET = gastric phase emulsion with propolis extract from Teotitlán de Flores Magón, GPESP = gastric phase emulsion with propolis extract from San Pedro Ocopetatillo, GPESJ = gastric phase emulsion with propolis extract from San Jerónimo Tecoalt, IPET = intestinal phase emulsion with extract of propolis from Teotitlán de Flores Magón, IPESP = intestinal phase emulsion with propolis extract from San Pedro Ocopetatillo, IPESJ = intestinal phase emulsion with propolis extract from San Jerónimo Tecoalt.

## Data Availability

The data presented in this study are available on request from the corresponding author (data are part of an ongoing study).
